# A C-type lectin in saliva of *Aedes albopictus* (Diptera: *Culicidae*) bind and agglutinate microorganisms with broad spectrum

**DOI:** 10.1093/jisesa/iead043

**Published:** 2023-07-03

**Authors:** Zimin Lin, Jinzhi Cheng, Xiaohui Mu, Xiaoyuan Kuang, Zhiqiang Li, Jiahong Wu

**Affiliations:** The Key and Characteristic Laboratory of Modern Pathogen Biology, College of Basic Medicine, Guizhou Medical University, Guiyang 550025, China; Department of Parasitology, Guizhou Medical University, Guiyang 550025, China; The Key and Characteristic Laboratory of Modern Pathogen Biology, College of Basic Medicine, Guizhou Medical University, Guiyang 550025, China; Department of Parasitology, Guizhou Medical University, Guiyang 550025, China; The Key and Characteristic Laboratory of Modern Pathogen Biology, College of Basic Medicine, Guizhou Medical University, Guiyang 550025, China; Department of Parasitology, Guizhou Medical University, Guiyang 550025, China; The Key and Characteristic Laboratory of Modern Pathogen Biology, College of Basic Medicine, Guizhou Medical University, Guiyang 550025, China; Department of Parasitology, Guizhou Medical University, Guiyang 550025, China; The Key and Characteristic Laboratory of Modern Pathogen Biology, College of Basic Medicine, Guizhou Medical University, Guiyang 550025, China; Department of Immunology, Guizhou Medical University, Guiyang 550025, China; The Key and Characteristic Laboratory of Modern Pathogen Biology, College of Basic Medicine, Guizhou Medical University, Guiyang 550025, China; Department of Parasitology, Guizhou Medical University, Guiyang 550025, China

**Keywords:** C-type lectin, agglutinating activity, Aedes albopictus, saliva, mosquito innate immunity

## Abstract

Via complex salivary mixture, mosquitos can intervene immune response and be helpful to transmit several viruses causing deadly human diseases. Some C-type lectins (CTLs) of mosquito have been reported to be pattern recognition receptor to either resist or promote pathogen invading. Here, we investigated the expression profile and agglutination function of an Aedes *albopictus* CTL (Aalb_CTL2) carrying a single carbohydrate-recognition domain (CRD) and WND/KPD motifs. The results showed that *Aalb_CTL2* was found to be specifically expressed in mosquito saliva gland and its expression was not induced by blood-feeding. The recombinant Aalb_CTL2 (rAalb_CTL2) could agglutinate mouse erythrocytes in the presence of calcium and the agglutinating activity could be inhibited by EDTA. rAalb_CTL2 also displayed the sugar binding ability to D-mannose, D-galactose, D-glucose, and maltose. Furthermore, it was demonstrated that rAalb_CTL2 could bind and agglutinate Gram positive bacteria *Staphylococcus aureus* and *Bacillus subtilis*, Gram negative bacteria *Escherichia coli* and *Pseudomonas aeruginosa*, as well as fungus *Candida albicans* in vitro in a calcium dependent manner. However, rAalb_CTL2 could not promote type 2 dengue virus (DENV-2) replication in THP-1 and BHK-21 cell lines. These findings uncover that Aalb_CTL2 might be involved in the innate immunity of mosquito to resist microorganism multiplication in sugar and blood meals to help mosquito survive in the varied natural environment.

## Introduction

The C-type lectins (CTLs), a Ca^2+^-dependent super family of proteins, have 1 or more carbohydrate recognition domains (CRD) and can regulate a diverse physiological function. According to their phylogeny and function, they are classified into 17 subgroups. Most CTLs contain only 1 carbohydrate recognition domain, which indicates that they can specifically bind to mannose, galactose, glucose, and other sugars (Zelensky and Gready 2005, Brown et al. 2018). According to the characteristics of CRD and carbohydrate binding ability, the classical CTLs include mannose specificity with EPN (Glu-Pro-Asn) motif and galactose specificity with QPD (Gln-Pro-Asp) motif (Zelensky and Gready 2005). In vertebrate, as 1 of the pattern recognition receptors (PRRs), CTLs can recognize pathogen-associated molecular patterns (PAMP) such as lipopolysaccharide (LPS), peptidoglycan (PGN), and flagellin, leading to trigger downstream immune responses (Zhu et al. 2013), which are essential in phagocytosis, agglutination, encapsulation, melanization, and prophenoloxidase activation (Wang et al. 2009a, 2009b, 2011, Zhao et al. 2009).

At present, many CTLs have been predicted in the genome of mosquito. A total of 52 CTLs have been predicted in *Aedes aegypti*, 55 in *Culex quinquefasdatus*, and 25 in *Anopheles gambiae* (Christophides et al. 2002, Bartholomay et al. 2010, Adelman and Myles 2018). Different CTLs have been implicated diverse physiological functions. An *Ae. aegypti* C-type lectin *CLSP2*, is demonstrated to restrain hemolymph melanization by inhibiting the activation of prophenoloxidase (PPO) (Shin et al. 2011). *AsCTLGA5*, a C-type lectin from *Armigeres subalbatus*, can significantly reduce the infection of *E. coli* (Shi et al. 2014). Two C-type lectins (*CTL4* and *CTLMA2*) in *An. gambiae* can resist gram-negative bacteria (Osta et al. 2004, Schnitger et al. 2009, Simões et al. 2022). However, some researches have also demonstrated that some CTLs can facilitate virus infection. In *Ae. aegypti* CTLs, *mosGCTL-1* can interact with West Nile virus to promote virus infection, and *mosGCTL-3* can contribute to Dengue virus (DENV) infection(Cheng et al. 2010, Liu et al. 2014).

Previous studies have shown that the salivary proteins of mosquito have multiple functions such as inhibiting blood coagulation, anti-inflammatory reaction, and promoting pathogen transmission (Calvo et al. 2011, Kaczmarek et al. 2020, Martin-Martin et al. 2020). With the rapid development of genomics and proteomics technology, a variety of mosquito salivary gland cDNA libraries have been constructed (Valenzuela et al. 2003, Calvo et al. 2004, 2007, 2010, Ribeiro et al. 2004, 2007, Arcà et al. 2005), which make it possible and easy to study the function of saliva proteins. Arcà (Arcà et al. 2007) constructed the salivary gland cDNA library of *Ae. albopictus* in 2007 and identified only 2 CTLs genes named as *Aalb_CTL1* and *Aalb_CTL2*. The rAalb_CTL1 has been proved to be a Ca^2+^-dependent C-type lectin that can specifically bind to mannose and agglutinate *Staphylococcus aureus* and *Candida albicans* (Cheng et al. 2014). However, the function of Aalb_CTL2 is less known.

In this study, to investigate the potential functions of *Ae. albopictus Aalb_CTL2*, we initially cloned and expressed this specifically expressed protein in salivary glands. We found that, in the presence of 40 mM Ca^2+^, rAalb_CTL2 could exert agglutinative function when co-cultured with mouse erythrocytes, and the agglutination activity could be inhibited by the sugars. And it also agglutinated bacteria and fungi that commonly exist in mosquito saliva. As for the role in mosquito-borne virus DENV-2 transmission, rAalb_CTL2 seems to have no contribution reflected by no effect on DENV-2 replication in THP-1 and BHK-21 cells. All of these suggested that Aalb_CTL2 might be involved in innate immunity to resist microbe infections in mosquito saliva.

## Materials and Methods

### Mosquito Maintenance, Microorganisms, Virus, and Cell Lines

*Ae. albopictus* (Guangzhou strain) used in this study were routinely maintained in our lab (Cheng et al. 2014). Gram-positive bacteria: *S. aureus* (ATCC25923), *B. subtilis* (ATCC6633), Gram-negative bacteria: *E. coli* (ATCC25922), *P. aeruginosa* (ATCC27853), and fungi: *C. albicans* (ATCC10231) were obtained from the Beijing Institute of Microbiology and Epidemiology and maintained in our laboratory. DENV-2, Baby hamster kidney cell (BHK-21) and human monocytic cell (THP-1) were stored in our laboratory. BHK-21 cells were maintained in Dulbecco’s modified Eagle’s medium (DMEM) (Gibco, USA) supplemented with 10% FBS, 100 U/ml penicillin, and 100μg/ml streptomycin sulfate at 37°C with 5% CO_2_. THP-1 cells were passaged in RPMI-1640 medium with 10% heat-inactivated FBS, 100 U/ml penicillin, and 100 μg/ml streptomycin sulfate at 37°C with 5% CO_2_.

### Sequence Analysis

*Aalb_CTL2* gene sequence was obtained from NCBI (https://www.ncbi.nlm.nih.gov/nuccore/AY826069.1). The protein sequences were deduced from the cDNA sequences using the ExPASy program (http://web.expasy.org/translate/). Signal peptide and CRD domain prediction were conducted using SMART software (http://smart.embl-heidelberg.de/).

### Tissue Distribution and Blood-Feeding Various Time Points Expression Profile of *Aalb_CTL2*

Tissue samples including salivary glands (SG), mid-guts (MG), and fat bodies (FB) from the sugar-fed adult female mosquitoes at 5 days old were dissected in the normal saline (NS:0.9% NaCl, pH 6.5) and stored in the TRIzol Reagent (Invitrogen, USA) at −80 °C. SGs at different blood-feeding time points were collected as previously described (Cheng et al. 2014). All the samples included 6 biological replicates, each consisted of salivary glands from 6 mosquitoes, midgut, and fat body from 1 mosquito.

Total RNA was extracted from SGs, MGs, and FBs of *Ae. albopictus* according to the TRIzol Reagent manufacturer’s protocols. Genomic DNA was removed and RNA was reversely transcribed to cDNA using RT reagent kit with gDNA Eraser (TaKaRa, #RR047A). Primers listed in Table 1 were used for cloning the mature peptide of *Aalb_CTL2* and detecting the expression of *Aalb_CTL2* and *rsp5* (ribosomal protein 5 gene as an internal control) genes by quantitative real-time PCR (qRT-PCR) (TB Green premix Ex TaqⅡ, #RR820A). Dissociation curve analysis of amplification products was performed. The expression level of *Aalb_CTL2* was evaluated by double standard curves method.

**Table 1.** The primers used in the present study

Primer	Forward primer (5ʹ–3ʹ)	Reverse primer (5ʹ–3ʹ)	Product length (bp)	Purpose
*Aalb_CTL2*	GACCATATGCAGGAGAAGTGTGATTCACAGAAC	CGCCTCGAGCTATTTTTTCTTTCTCTCACACACG	411	cloning
*Aalb_CTL2*	GCTTGCCAGGAGAAGTGTGAT	ACTGTTGACCACGGCCAGTT	120	qRT-PCR
*Rsp5*	ATTACATCGCCGTCAAGGAG	TCATCATCAGCGAGTTGGTC	126	qRT-PCR
*E-DENV*	CAGATCTCTGATGAATAACCAACG	CATTCCAAGTGAGAATCTCTTTGTCA	121	qRT-PCR
*β-actin*	TGACGTGGACATCCGCAAAG	CTGGAAGGTGGACAGCGAGG	205	qRT-PCR

The underline means restriction digestion enzyme site.

### Cloning, Expression, and Purification of Recombinant *Aalb_CTL2*

Specific primers (see Table 1) for mature peptide of *Aalb_CTL2* were designed according to the published sequence (Accession No. AY826069.1). The PCR product was cloned into the pET-28a(+) expression vector by double digest with restriction enzymes *Nde*Ⅰ/*Xho*Ⅰ(TaKaRa, Japan) and transformed into *E.coli* BL21 (DE3). Then the positive clones were induced to express using 1 mM isopropyl-β-d-thiogalactopyranoside (IPTG) at 37 °C for 5 h in LB medium (200 rpm/min, 37 °C). The cells were harvested by centrifugation at 12,000 rpm for 5 min and sonicated, and the inclusion bodies were resuspended in 1× phosphate-buffered saline (PBS) containing 8M urea. Recombinant Aalb_CTL2 (rAalb_CTL2) protein was refolded using a linear urea solution (contain 6, 5, 4, 3, 2, 1, 0.5, and 0 M) gradient in dialysate at 4 °C overnight. rAalb_CTL2 was purified by His Band Purification Kit (Novagen) according to the manufacturer’s instructions. Purified recombinant protein was dialyzed against 1× PBS buffer at 4 °C overnight and was verified by 15% sodium dodecyl sulfate-polyacrylamide gel electrophoresis (SDS-PAGE).

### Hemagglutination Assay of rAalb_CTL2

The mouse erythrocytes were collected from mice and prepared to be used after being washed 5 times with normal saline. About 12.5 μl of rAalb_CTL2 with a serial 2-fold dilution from 60 μg/ml to 0.93 μg/ml were mixed with different concentrations (0, 10, 20, 30, 40, 50, 60, 70, 90, 100, 200 mM) of CaCl_2_ at a final volume of 25 μl. Finally, 25 μl of 2% mouse erythrocytes were added, and the mixture was incubated at 25 °C for 30 min. Hemagglutination of erythrocytes was observed by naked eyes. The assay was performed in triplicate.

### Sugar Binding Specificity Assay of rAalb_CTL2

Lectins exert the agglutinative function via binding certain sugars. In this assay, 12.5 μl of serial dilutions of various carbohydrates including d-mannose, d-galactose, d-glucose, and maltose (from 1 M to 0.195 mM) in normal saline containing 40 mM of Ca^2+^ were mixed with 12.5 μl of rAalb_CTL2 (7.5 μg/ml) and incubated at 37 °C for 30 min. Then, 25 μl of 2% mouse erythrocytes suspension were added and incubated at room temperature for 30 min. Finally, an inhibitory effect was evaluated by the minimum sugar concentration that was shown to completely inhibit agglutinating activity of the rAalb_CTL2. This experiment was performed in triplicate independently.

### Microbial Agglutination Assay of rAalb_CTL2

The microorganisms (*S. aureus*, *B. subtilis*, *E. coli*, *P. aeruginosa*, and *C. albicans*) in the mid-logarithmic phase were resuspended in tris-buffered saline (TBS) (with or without 40 mM CaCl_2,_ or EDTA plus 40 mM CaCl_2_), and stained with 4,6-diamidino-2-phenylindole (DAPI) (Thermofisher, #62248). Then the DAPI-stained microorganisms were incubated with rAalb_CTL2 (50 μg/ml) at room temperature for about 20 min. At last, the microbial agglutination was observed by fluorescence microscopy.

### Microbial Binding Assay of rAalb_CTL2

The microorganisms (*S. aureus*, *B. subtilis*, *E. coli*, *P. aeruginosa*, and *C. albicans*) in the mid-logarithmic phase were suspended in TBS (25 mM Tris–HCl, 137 mM NaCl, and 3 mM KCl, pH7.0) and incubated with rAalb_CTL2 (50 μg/ml) for 20 min at 37 °C. After washing 3 times with TBS, the microorganisms were incubated with rat polyclonal antibody againstrAalb_CTL2 (self-made) for 2 h at room temperature. Then they were washed 3 times with TBS again, and incubated with FITC-labeled anti-rat secondary antibodies (Absin, #CL36131) for 1 h at ambient temperature in the dark. Finally, the microorganisms were stained with DAPI at room temperature for 20 min, the results were imaged by confocal laser scanning microscopy.

### Detection of Dengue Virus Replication

THP-1 cells (2 × 10^5^ cells/well) were seeded in a 48-well plate for 12 h and then incubated with different concentrations (0.01, 0.1, 1 μg/ml) of rAalb_CTL2 mixed with DENV-2 (0.25 MOI) for 24 h. BHK-21 cells (1.25 × 10^5^) were cultured in a 24-well plate for 12 h and were treated with 2 μg/ml rAalb_CTL2 or inactivated rAalb_CTL2 (heat treatment at 100 °C for 15 min) mixed with DENV-2 (2 MOI) for 24 h. Total RNA was isolated and reversely transcribed into cDNA to detect DENV-2 envelope gene expression. qRT-PCR was performed on ABI 7300 as followed: 95 °C for 5 min, 40 cycles of 95 °C for 10 s, 60 °C for 34 s. The target gene messenger RNA levels were normalized to human β-actin RNA levels according to 2^−ΔΔ^Ct calculations. The qRT-PCR primers sequences are listed in Table 1.

### Statistical Analysis

Significant difference was determined by the unpaired t-test or one way ANOVA followed by a Tukey’s multiple comparison test (GraphPad, San Diego, CA). The quantification was calculated based on the cycle threshold (Ct) value generated by qRT-PCR. All data obtained is presented as the means ± SD from 3 independent experiments. *P* values ≤ 0.05 represents significant differences between compared groups.

## Results

### The mRNA Expression Pattern of *Aalb_CTL2* in Tissues and Different Blood-Feeding Stages

**Fig. 1.**
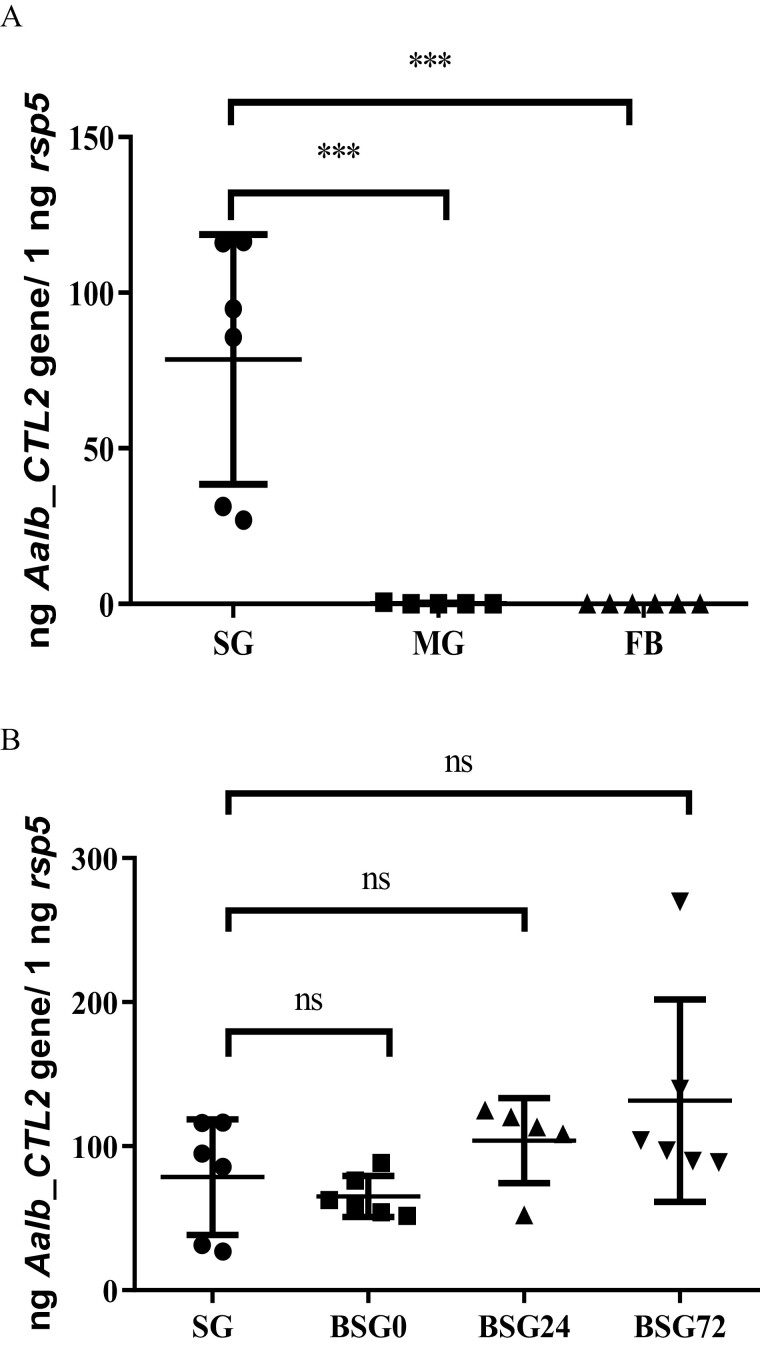
mRNA expression pattern of *Aalb_CTL2* in tissues and different blood-feeding stages. A) The mRNA levels of *Aalb_CTL2* in 5-day-old female *Ae. albopictus* salivary gland (SG), mid-gut (MG), and fat body (FB) (*n* = 6). B) The mRNA levels of *Aalb_CTL2* in female *Ae. albopictus* salivary gland at different blood-feeding stages. ***P < 0.001.

The mRNA expression pattern of *Aalb_CTL2* in 3 different female mosquito parts was detected using qRT-PCR. The result was shown in Fig. 1. The mRNA level of *Aalb_CTL2* was much higher in salivary glands than that in midgut and fat body, indicating Aalb_CTL2 would be an important protein component in mosquito saliva (Fig. 1A). And there was no significant difference in the expression level of *Aalb_CTL2* during different blood-feeding stages (Fig. 1B).

### Sequence Analysis of *Aalb_CTL2*

**Fig. 2.**
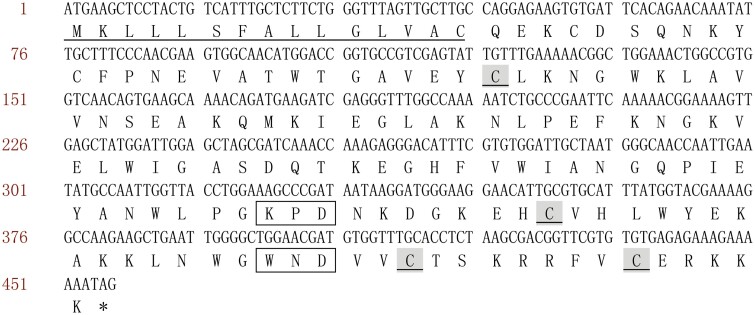
ORF and deduced amino acid sequence of *Aalb_CTL2*. The amino acid sequence is derived from the nucleic acid sequence. The termination codon is indicated by an asterisk (*). The signal peptide sequence is underlined, 4 conservative cysteine residues are marked with gray shading, and the sugar-specific recognition site KPD motif and calcium binding site WND motif are in box.

The cDNA sequence of *Aalb_CTL2* had an open reading frame (ORF) of 456bp encoding 151 amino acids with a 15 amino acid N-terminal signal peptide. The calculated molecular mass of the mature Aalb_CTL2 protein (residues 16 to 151) was 15.71 kDa, with an estimated pI of 9.0. Aalb_CTL2 had a single characteristic CRD domain with 4 conserved Cys residues at position 41,118,138 and 146. There was a Ca^2+^-binding site known as “WND” (Trp-Asn-Asp) motif and galactose-specific carbohydrate-binding motif “KPD” (Lys-Pro-Asp) (Fig. 2).

### The Recombinant Protein of Aalb_CTL2

**Fig. 3.**
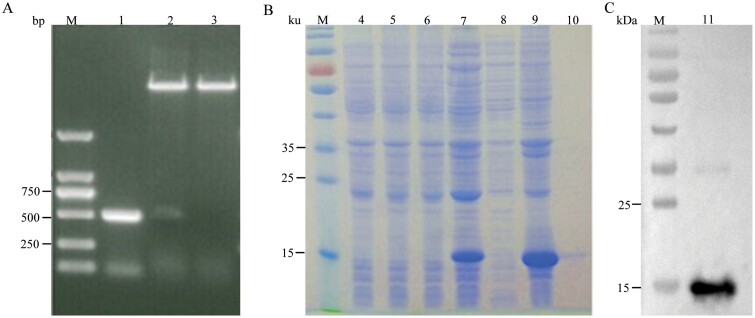
Cloning, expression, and purification of recombinant Aalb_CTL2 protein. A) Detection of PCR products and enzyme digestion identification of recombinant plasmid pET-28a-Aalb_CTL2, M: DL 2000 Marker, 1: *Aalb_CTL2* PCR amplification product; 2: pET-28a-Aalb_CTL2 after double enzyme digestion; 3: pET-28a-Aalb_CTL2 after single enzyme digestion. B) Expression and purification of rAalb_CTL2 in *E. coli* BL21(DE3) M: standard protein marker; 4: Total protein before empty plasmid induction; 5: Total protein after induction of empty plasmid; 6: Total protein before induction of recombinant plasmid; 7: Total protein induced by recombinant plasmid; 8: Induced supernatant; 9: Induced deposit; 10: The purified rAalb_CTL2. C) Western blotting identification of rAalb_CTL2, 11: anti-his-tag; M: standard protein marker.

To investigate the potential function of Aalb_CTL2, a recombinant plasmid pET-28a-*Aalb_CTL2* was constructed and expressed successfully in *E.coli* BL21 (D3). A distinct band with a molecular weight about 15 kDa was detected by SDS-PAGE (Fig. 3).

### Hemagglutination Activity of rAalb_CTL2

**Fig. 4.**
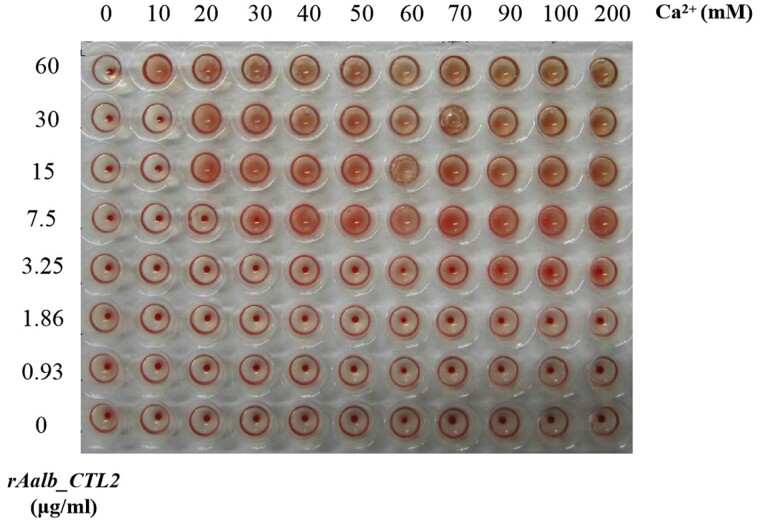
Agglutination activity of rAalb_CTL2 on mouse red blood cells at a Ca^2+^-dependent manner. The mouse erythrocytes were added into 96 V-shape plates and incubated with rAalb_CTL2 and Ca2+ at 25 °C for 30 min.

As shown in Fig. 4, the hemagglutination activity increased with the addition of different concentrations of rAalb_CTL2 in the presence of Ca^2+^, which suggests the agglutination activity of rAalb_CTL2 was Ca^2+^dependent, and the optimal concentration of Ca^2+^ is 40mM.

### Carbohydrate Binding Specificity of rAalb_CTL2

To detect the carbohydrate binding ability of rAalb_CTL2, we investigated the inhibitory activity of rAalb_CTL2 for sugar binding. As shown in Table 2, in the presence of 40 mM Ca^2+^, the agglutinating activity of rAalb_CTL2 was inhibited by all 4 types of sugars, d-Glucose, d-Mannose, d-Galactose, and Maltose at ­different concentrations. Based on the minimum inhibitory concentration, the sugar inhibitory activity was evaluated to be Maltose < d-Glucose < d-Galactose < d-Mannose.

**Table 2.** Inhibition of agglutination of Aalb_CTL2 by sugars

Sugar	Minimum inhibitory concentration (mM)
d-Glucose	12.5
d-Mannose	3.125
d-Galactose	6.25
Maltose	25

### Microbial Agglutination Capacity of rAalb_CTL2

**Fig. 5.**
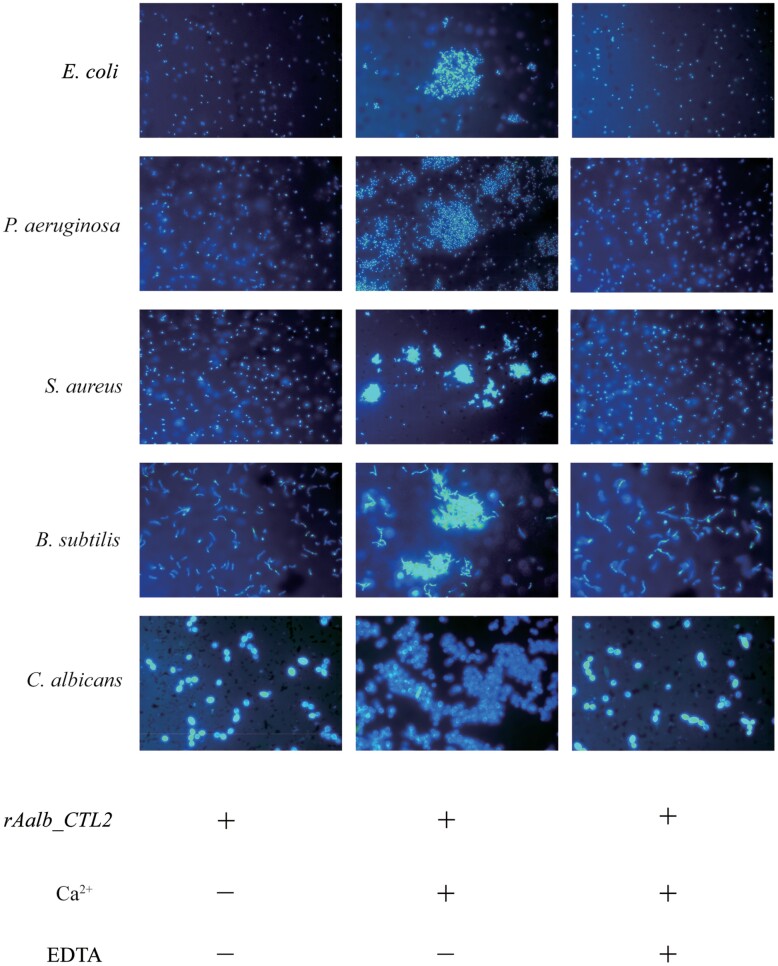
rAalb_CTL2 induced the agglutination to microbes in a Ca2+ dependent manner. (×40) Nuclei of microorganism was stained by DAPI.

C-Type lectin can recognize the sugar molecular structure on the surface of bacteria and cause bacterial agglutination. To further investigate whether rAalb_CTL2 could agglutinate bacteria, a microbe agglutinating assay (*S. aureus*, *B. subtilis*, *E. coli*, *P. aeroginosa*, and *C. albicans*) was performed. As shown in Fig. 5, rAalb_CTL2 significantly agglutinated all the 5 microbes in the presence of Ca^2+^. When a calcium-chelating agent EDTA was added, the aggregation of bacteria was inhibited. It indicated that the agglutinating activity of rAalb_CTL2 was at a Ca^2+^-dependent manner. All the 5 microbes could be agglutinated by rAalb_CTL2 which suggesting that rAalb_CTL2 had a broad agglutinating activity on microbes (Fig. 5).

### Microbial Binding Assay of rAalb_CTL2

**Fig. 6.**
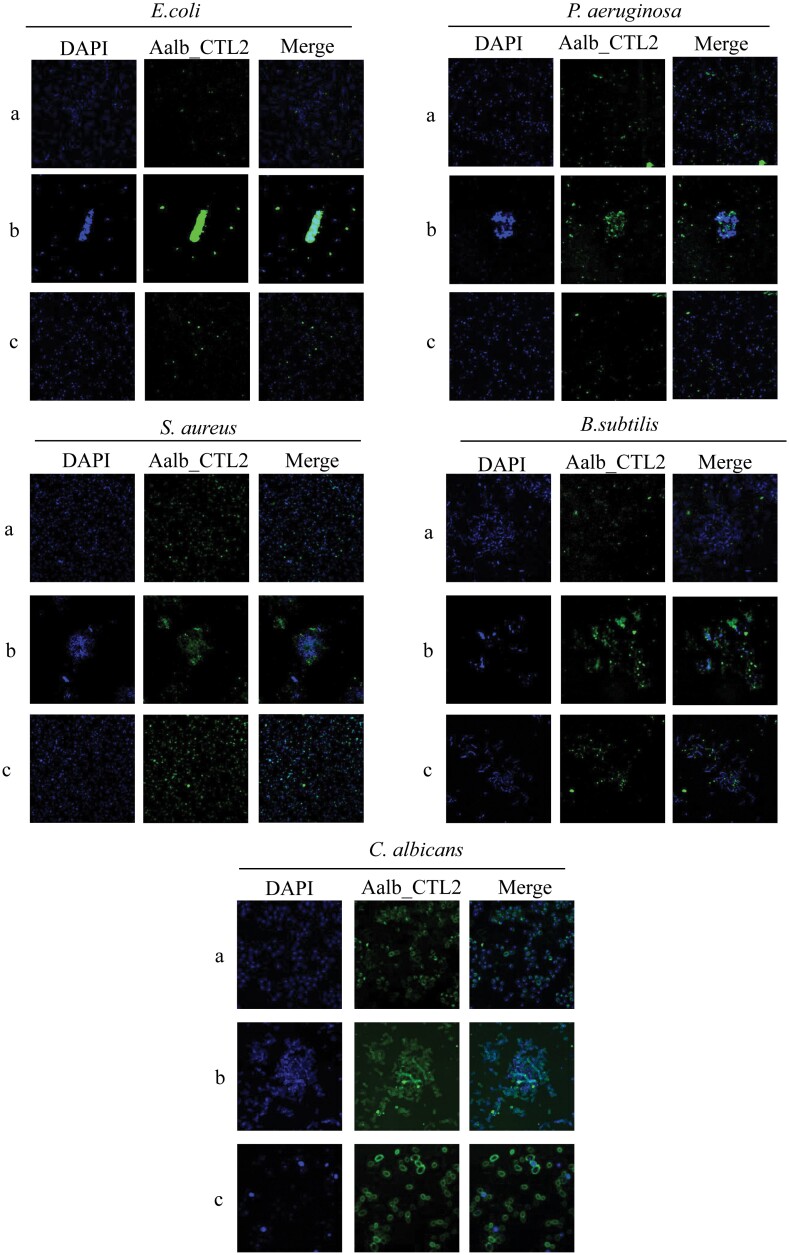
Analysis of binding affinity with microorganisms of rAalb_CTL2 by IFA. rAalb_CTL2 was identified by rat polyclonal antibodies against rAalb_CTL2 (in green); Nuclei of microorganism was stained by DAPI (in blue). A) The bacteria incubated with rAalb_CTL2. B) The bacteria incubated with rAalb_CTL2 and in the presence of 40 mM Ca^2+^. C) The bacteria incubated with rAalb_CTL2 and in the presence of 40 mM Ca^2+^ and EDTA. The agglutinate bacteria were viewed at 20× magnification and others were viewed at 40× magnification.

To determine how rAalb_CTL2 agglutinated microorganisms, we detected whether the rAalb_CTL2 bound them by Indirect Immunofluorescence Assay (IFA). As we expected, the rAalb_CTL2 could agglutinate all the microorganisms in the presence of Ca^2+^, and rAalb_CTL2 had binding ability to the surface of the microorganisms. The agglutination of all microorganisms disappeared either with EDTA or without Ca^2+^, however, rAalb_CTL2 still could bind on the surfaces of *S. aureus* and *C. albicans* (Fig. 6).

### rAalb_CTL2 did not Promote DENV-2 Replication

**Fig. 7.**
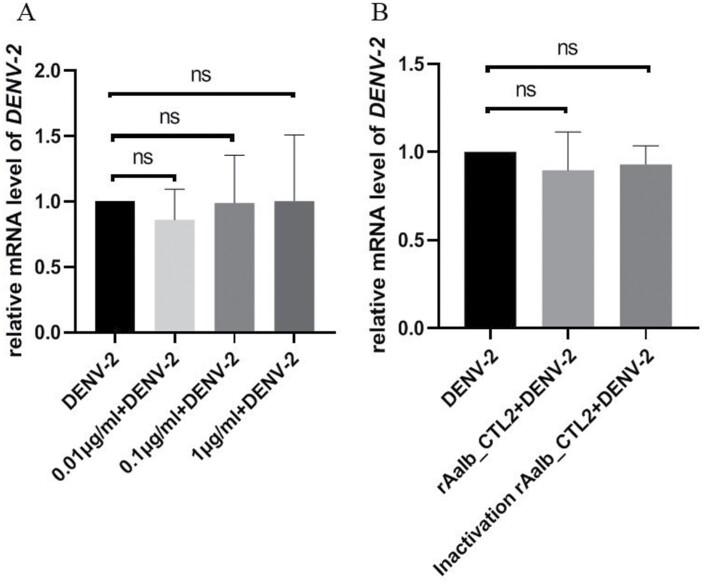
rAalb_CTL2 could not promote DENV-2 replication in THP-1 and BHK-21 cell lines. A) The relative mRNA level of *E* gene in DENV-2 infected THP-1 cell line with different concentration of rAalb_CTL2 at 24 h (MOI = 0.25, *n* = 6). B) The relative mRNA level of *E* gene in DENV-2 infected BHK-21 cell line with 2 μg/ml rAalb_CTL2 and 2 μg/ml inactivated rAalb_CTL2 at 24 h (MOI = 2, *n* = 6).

As an important protein component of mosquito saliva, we further investigated the virus replication when THP-1 and BHK-21 cell lines were challenged with DENV-2 alone or the mixture of rAalb_CTL2 and DENV-2. Compared with the DENV-2 alone challenge group, the DENV-2 groups challenging with different concentrations of rAalb_CTL2 showed no significant differences on the mRNA level of E gene in both THP-1 and in BHK-21 cell lines (Fig. 7).

## Discussion

CTLs have been identified widely in various insects as a result of whole genome sequencing. In mosquito, as important PRRs, some CTLs have been found to play significant roles in innate immunity against microbes. However, some CTLs have also been found to promote pathogen infection (Xia et al. 2018). *Aalb_CTL2* is 1 of only 2 CTLs identified in transcriptome of salivary gland of *Ae. albopictus*. It has a signal peptide which means it can be secreted into saliva and could enter into host skin with saliva. Therefore, we cloned and expressed rAalb_CTL2, and the possible biological function was investigated in this study.

The rAalb_CTL2 could agglutinate mouse erythrocytes at 40 mM CaCl_2_ and the agglutination activity could be inhibited by EDTA, which indicated rAalb_CTL2 was a typical C-type lectin. It is worth mentioning that the rAalb_CTL2 demonstrated a wide sugar binding spectrum by binding not only galactose but also D-mannose and other sugars, although it had a KPD motif indicating galactose specificity. In fact, many studies have shown the motif of invertebrate CTLs is conservative but not the function (Pees et al. 2016). The C-type lectin *PtCLec2* from *Portunus trituberculatus* with a QPD motif, which is predicted to bind galactose, but has also been shown to bind not only galactose, but also d-mannose and sucrose in vitro (Liu et al. 2021). *OmLec1*, the C-type lectin of the *Onychostoma macrolepis*, is predicted to have a mannose-specific binding motif ENP, but can also bind d-glucose, PGN, and LPS (Shang-Guan et al. 2021b).

As pattern recognition receptors, an important feature of CTLs is that they can recognize and bind the polysaccharides on the surface of pathogens, which can produce agglutination reaction by binding with glycoconjugates on the cell surface and participate in innate immunity in vivo and in vitro (Wang et al. 2018, Bi et al. 2020, Shang-Guan et al. 2021a). A C-type lectin DL1 has been purified from a pupal extract of *Drosophila meianogaster*, can agglutinate with *E. coli* and *Erwinia chrysanthemi* (Tanji et al. 2006), while the other 2 lectins DL2 and DL3 in *Drosophila melanogaster* can agglutinate *E. coli* in the presence of Ca^2+^(Ao et al. 2007). These results demonstrate that different lectins have different agglutinating activity. In this study, rAalb_CTL2 exhibited a wide microbes agglutinated spectrum, including the gram-positive bacteria (*S. aureus* and *B. subtilis*), gram-negative bacteria (*E. coli* and *P. aeruginosa*), and fungi (*C. albicans*). While, rAalb_CTL1, another CTL specifical expressed in female salivary gland of *Ae. albopictus*, can only agglutinate *C. albicans* and *S. aureus*, but not *E. coli* in vitro (Cheng et al. 2014). The difference of bacteria agglutinating activity might be related to their different carbohydrate binding capacity. Furthermore, we found that rAalb_CTL2 could bind to the surface of microorganisms in the presence of Ca^2+^, which might indicate that rAalb_CTL2 exert the agglutination function by combining with the surface of bacteria to resist invasion. In addition, it is interesting to find rAalb_CTL2 could still bind *S. aureus* and *C. albicans* even without Ca^2+^ but no agglutination. The similar result has been found in *Ms*IML-2 from *M. sexta* (Yu and Ma 2006). These results suggest that binding of some insect CTLs to ligands may not require calcium, but calcium binding may facilitate formation of lectin oligomers for agglutination. Of course, the different agglutinating microbial activity of Aalb_CTL1 and Aalb_CTL2 in saliva of *Ae. albopictus* means that there is a redundant function in mosquito saliva involved in controlling bacterial growth in sugar and blood meal, which help mosquito hosts to live in the environment.

As mentioned before, mosquito saliva plays an important role in arthropods-disease transmission, and also some researches demonstrated that some CTLs of mosquito could promote pathogen infection (Liu et al. 2014, Jin et al. 2018, Uraki et al. 2019). Therefore, we examined the effect of rAalb_CTL2 on DENV-2 replication in vitro. The results showed the rAalb_CTL2 could not promote the replication of DENV-2 in THP-1 and BHK-21 cell lines. The similar results have also been found in the rAalb_CTL1 (unpublished data). These results further demonstrated that both of the CTLs in mosquito saliva might mainly contribute to mosquito innate immunity.

In conclusion, Aalb_CTL2, a C-type lectin specifically expressed in salivary glands of *Ae. albopictus*, was cloned and expressed in *E. coli*. The rAalb_CTL2 demonstrates a typical agglutinating activity of erythrocyte in a calcium dependent manner and has a wide spectrum of sugar binding capacity. More importantly, it cannot promote DENV-2 replication in mammal cell lines but exhibit a broader microorganisms agglutination profile toward Gram-positive bacteria *S. aureus*, *B. subtilis*, Gram-negative bacteria *E. coli*, *P. aeroginosa*, as well as fungus *C. albican* in a calcium dependent manner. It is also found that rAalb_CTL2 has binding activity only with *S. aureus* and *C. albican* without calcium. The results suggest that *Aalb_CTL2*, as a PRR, might be involved in the innate immunity of mosquito to resist microorganism multiplication in sugar and blood meal, which helps mosquito to survive in the environment. In addition, our findings also provide new insights into the function of saliva protein in *Ae. albopictus.*
